# Effects of Different Rootstocks on Graft Compatibility, Growth, Yield, and Fruit Quality of Table Grape ‘Fengguang’

**DOI:** 10.3390/plants14193098

**Published:** 2025-10-08

**Authors:** Nan Jia, Minmin Li, Changjiang Liu, Bin Han, Yan Sun, Shuli Han, Xinyu Wang, Yonggang Yin

**Affiliations:** Changli Institute of Fruit Research, Hebei Academy of Agriculture and Forestry Sciences, Qinhuangdao 066000, China

**Keywords:** *Vitis vinifera* L., grafting, vigor, principal components analysis, comprehensive evaluation

## Abstract

Selecting an appropriate rootstock for a specific scion cultivar is an efficient way to improve both yield and berry quality in viticulture. This study aimed to provide practical guidance for rootstock selection in the cultivation of the table grape cultivar ‘Fengguang’. The mature scions of this cultivar were grafted onto hardwood cuttings of eight different resistant rootstocks, which included 101-14M, 110R, 188-08, 3309C, 5BB, 5C, SO4, and Beta, with the own-rooted vines as control. Graft compatibility, growth vigor, yield performance, and fruit quality were compared and analyzed among the different grafting combinations. The results suggested that vines on 101-14M, 5BB, and Beta obtained higher germination rates of scions, better healing rates of the mating interface, and greater generation rates of root callus. Among these, vines on 5BB exhibited the largest scion trunk cross-sectional diameter. Furthermore, SO4 demonstrated the most significant improvement in yield, with an average increase of 13.54% compared to the control. Regarding berry quality, 101-14M significantly enhanced berry mass, pressure resistance, and flesh firmness relative to the controls, with average improvements of 7.67%, 11.34%, and 29.86%, respectively. Based on a comprehensive evaluation of yield and fruit quality indicators, 101-14M gained the highest value. In conclusion, 101-14M is preferentially recommended for grafting cultivation of ‘Fengguang’ vines. These findings could provide practical guidance for the cultivation of table grape cultivars.

## 1. Introduction

Grape (*Vitis vinifera* L.) is one of the world’s most important economic fruit trees, highly valued by consumers for unique tastes and rich nutritional profiles [1,2]. While grapes are primarily used for winemaking worldwide, table grapes account for the largest share of China’s grape market with a share exceeding 85% of total consumption [3,4,5]. In recent years, the fresh grape industry has gradually shifted toward high-quality and specialty-focused development driven by consumers’ growing demand for superior berry attributes. Grafting technology, as a key viticultural practice, plays a significant role in enhancing the physiological performance and economic traits of scion cultivars by leveraging the stress resistance, adaptability, and growth regulatory capabilities of rootstocks [6,7,8,9]. However, the success of grafting fundamentally relies on grafting compatibility—a concept whose real meaning extends far beyond the physical union of tissues. True compatibility represents the establishment of a functional physiological and molecular continuum between rootstock and scion, enabling efficient translocation of water, nutrients, hormones, and genetic signals [10,11,12]. It is this seamless integration that ensures long-term vine health, sustained vigor, and consistent yield, underscoring its critical importance in viticulture. When compatibility is compromised, it often leads to vascular dysfunction, metabolic imbalance, and even graft failure, severely limiting the potential benefits of rootstock use. Consequently, optimizing rootstock–scion combinations to achieve synergistic effects of high graft affinity, vigorous growth and premium-quality productivity has become a critical research focus in grapevine cultivation physiology.

‘Fengguang’ is a table grape cultivar independently bred in China [13]. Currently, this cultivar is grown in major grape production regions of North China. However, in commercial production, ‘Fengguang’ grapes frequently encounter critical challenges, including non-targeted rootstock selection, unstable graft compatibility leading to premature vine senescence, significant yield reduction, and inconsistent fruit quality parameters. Currently, research on grapevine rootstock–scion interactions are primarily focused on wine grape cultivars (e.g., ‘Cabernet Sauvignon’ [14,15], ‘Merlot’ [16,17,18],‘Petit Verdot’ [19], ‘Marselan’ [20]) and conventional table grape cultivars (e.g., ‘Shine Muscat’ [21,22], ‘Summer Black’ [23,24], ‘Red Globe’ [25,26], ‘Kyoho’ [27,28]), with an emphasis on berry quality-related phenotypic traits. Key findings demonstrate that rootstock selection significantly modulates critical berry quality traits in a cultivar-specific manner. For instance, the SO4 rootstock exerted contrasting effects on anthocyanin concentrations, decreasing them in ‘Cabernet Sauvignon’ [14] but increasing them in ‘Merlot’ [16]. Rootstocks also markedly influence sugar accumulation (e.g., 101-14M produced the highest soluble solids in ‘Marselan’ [20]) and aroma profiles (e.g., 3309C elevated the total aroma in ‘Shine Muscat’ [22]). In contrast, attributes like the specific sugar composition in ‘Summer Black’ [23] and the acidity in ‘Red Globe’ [25] are predominantly scion-inherent, showing minimal plasticity across rootstocks. As a promising new table grape cultivar with considerable market potential, systematic evaluations of multiple rootstock–scion combinations for ‘Fengguang’ vines remain critically underexplored.

Therefore, ‘Fengguang’ vines were grafted onto eight commonly used rootstocks with different resistance and growth vigor, with own-rooted vines (non-grafted) as the control. The impacts of different scion–rootstock combinations on graft compatibility, vine vigor, yield, and berry quality were systematically evaluated. This study aimed to provide a scientific basis for selecting optimal rootstocks for ‘Fengguang’ vines production. Furthermore, this research contributed to the theoretical framework of scion–rootstock interactions and quality regulation in the field of fruit tree grafting physiology, providing valuable insights for the transformation and upgrading of the grape industry.

## 2. Results

### 2.1. Graft Compatibility Indicators

**Figure 1:**
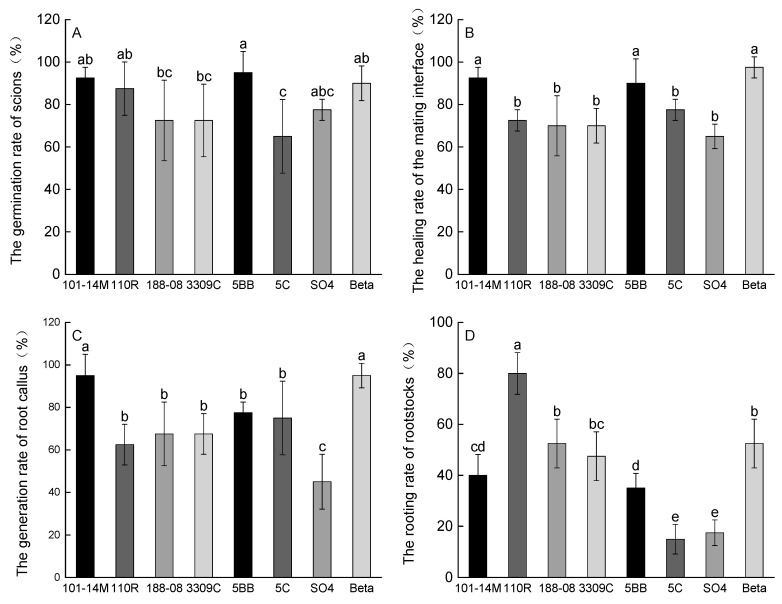
Impacts of different rootstocks on graft compatibility indicators of ‘Fengguang’ vines. Different lowercase letters within each figure represent significant differences at *p* < 0.05 by Duncan’s test. (**A**) The germination rate of scions; (**B**) The healing rate of the mating interface; (**C**) The generation rate of root callus; (**D**) The rooting rate of rootstocks.

Rootstocks had a significant effect on graft compatibility (Figure 1). The highest germination rate of scions was observed in vines on 5BB, while the lowest was observed in those on 5C (Figure 1A). Vines on 101-14M, 5BB, and Beta exhibited a significantly higher healing rate of the mating interface than those on the other five rootstocks (Figure 1B). The generation rate of root callus also varied significantly among rootstocks, with 101-14M and Beta showing the highest rates and SO4 the lowest (Figure 1C). The root rooting rate was highest in 110R and lowest in 5C and SO4 (Figure 1D).

### 2.2. Vine Growth

**Figure 2:**
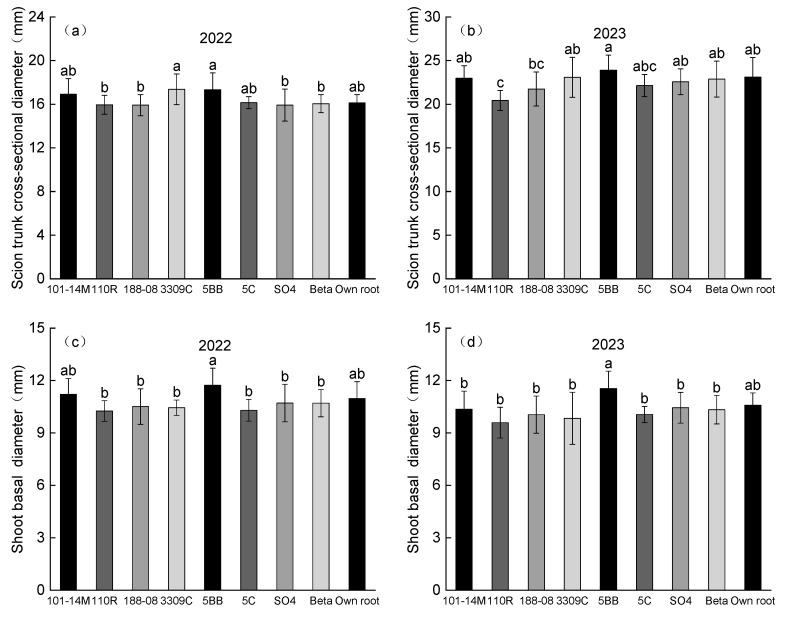
Impacts of different rootstocks on vine growth indicators of ‘Fengguang’ vines. Different lowercase letters within each figure represent significant differences at *p* < 0.05 by Duncan’s test. (**a**) Scion trunk cross-sectional diameter (2022); (**b**) Scion trunk cross-sectional diameter (2023); (**c**) Shoot basal diameter (2022); (**d**) Shoot basal diameter (2023).

As expected, scion trunk cross-sectional diameters (TCSDs) in 2023 were higher than those in 2022 (Figure 2a,b). In 2022, vines on 5BB and 3309C gained the highest scion TCSD, and no significant differences were observed among the graft combinations and the own-rooted vines (Figure 2a). In 2023, vines on 110R exhibited a significantly lower scion TCSD than that of own-rooted vines, while no significant difference was detected between vines on other rootstocks and own-rooted vines (Figure 2b). Regarding shoot basal diameter, no significant differences were observed among rootstocks compared to own-rooted vines in 2022, although vines on 5BB had the highest value (Figure 2c). Additionally, the shoot basal diameter showed a similar pattern in 2022 and 2023 (Figure 2c,d).

### 2.3. Yield and Appearance Attributes of Cluster and Berry

Rootstocks significantly influenced yields (Table 1). In both 2022 and 2023, vines on SO4 exhibited significantly higher yields than own-rooted vines, with average increases of 13.54%, whereas those on 101-14M showed significantly lower yields, with an average reduction of 8.74%. No significant differences in yield were observed for other scion–rootstock combinations compared to own-rooted vines. Additionally, yields in 2023 were higher than those in 2022.

The external quality attributes of clusters and berries were also impacted by rootstocks (Table 1). In 2022, Vines on SO4 displayed significantly heavier clusters than own-rooted vines, whereas 101-14M and 3309C showed significantly lighter clusters. In 2023, no significant differences in cluster mass were detected between rootstock-grafted vines and own-rooted vines, though cluster mass was generally higher in 2023 than in 2022. Regarding berry characteristics, 101-14M and 5C significantly increased the berry mass compared to own-rooted vines in both years, whereas SO4, 3309C, 188-08, and 5BB significantly reduced it. Notably, 101-14M showed the highest average increase (7.67%) in berry mass compared to own-rooted vines across both years. For berry vertical diameter (BV), 101-14M showed a significant increase relative to own-rooted vines in 2022, while SO4 exhibited a significant decrease in 2023. The berry horizontal diameter (BH) of vines on 188-08 was significantly smaller than that of own-rooted vines in 2022. Additionally, 188-08 significantly reduced BH and increased the berry shape index (calculated as the ratio of BV/BH) in 2022. Notably, berry mass, BH, and BV were consistently lower for all rootstocks in 2023 than in 2022.

### 2.4. Physical and Chemical Indicators

Rootstocks exerted a significant influence on the physical and chemical indicators of fruits (Table 2). In both 2022 and 2023, vines on SO4 exhibited significantly lower pulling resistance compared to own-rooted vines, whereas other rootstocks showed no significant differences from own-rooted vines in either year, except for 188-08 in 2023. 101-14M and 5C significantly enhanced the pressure resistance in both years, while SO4 significantly decreased it relative to own-rooted vines. Similarly, compared to own-rooted vines, SO4 significantly reduced the flesh firmness, whereas 101-14M and 188-08 significantly improved it in both years. Specifically, compared to own-rooted vines, 101-14M provided the greatest enhancement in both pressure resistance and flesh firmness across the two years, with average increases of 11.34% and 29.86%, respectively; conversely, SO4 showed the largest reduction in these traits, with average decreases of 5.62% and 14.77%. Regarding sugar-related traits, Beta, 5C, SO4, and 5BB significantly lowered the total soluble solids (TSS) content relative to own-rooted vines in both years. For titratable acidity (TA), 5BB and Beta showed a significant increase in 2022, and SO4 exhibited a significant rise in 2023 compared to own-rooted vines. Furthermore, the TSS/TA ratio was significantly reduced by Beta, 5BB, and SO4 in both years compared to own-rooted vines.

### 2.5. Principal Component Analysis

**Figure 3:**
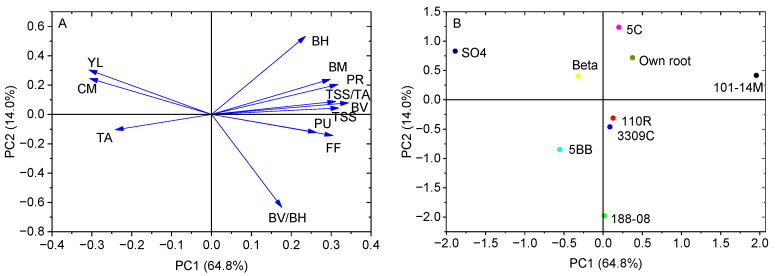
Principal component analysis of different rootstocks on yield and berry quality of ‘Fengguang’ grapes. (**A**) Loadings plot; (**B**) Score plot. YL, Yield; CM, cluster mass; BM, berry mass; BV, berry vertical diameter; BH, berry horizontal diameter; PU, pulling resistance; PR, pressure resistance; FF, flesh firmness; TSS, the total soluble solids; TA, titratable acid.

Principal components analysis (PCA)was performed on the dataset of 12 traits across 9 different graft combinations (including own-rooted vines) to provide us with an overview visualization in a reduced dimension (Figure 3). The first two PCs accounted for 78.8% of the total variation.

PC1 represented 64.8% of the variance and was positively correlated with BV, TSS/TA, TSS, pressure resistance (PR), flesh firmness (FF) and pulling resistance (PU), while exhibiting negative correlations with cluster mass (CM) and TA (Figure 3A). Additionally, PC2 explained 14.0% of the total variation and was characterized by a positive correlation with BH and a negative correlation with the berry shape index (BV/BH) (Figure 3A). Yield (YL) contributed substantially to both PCs, showing a negative correlation with PC1 and a positive correlation with PC2 (Figure 3A).

Different scion–rootstock combinations were clearly separated by the first two PCs (Figure 3B). PC1 effectively distinguished vines on 101-14M from those on other rootstocks, particularly from SO4. (Figure 3B). Vines on 101-14 exhibited higher values of BV, TSS/TA, TSS, PR, FF and PU and lower values of CM and TA. Vines on SO4 showed superiority in improving YL, CM, and TA. Meanwhile, a higher value of BV/BH was obtained by vines on 188-08.

### 2.6. Comprehensive Evaluation of Yield and Berry Quality

To comprehensively evaluate the effects of different scion–rootstock combinations on yield and quality traits, a membership function method was applied for integrated assessment (Table 3). The two highest comprehensive scores were achieved by 101-14M and own-rooted vines, indicating their decisive superiority in quality-related characteristics. Conversely, SO4 ranked last in the overall evaluation. Although SO4 could significantly increase yield and cluster weight, it was associated with comparatively lower performance in quality-related traits. The remaining rootstocks occupied intermediate positions within the overall ranking.

## 3. Discussion

Grafting affinity is a fundamental criterion for evaluating the quality of scion–rootstock combinations. In this study, vines on 101-14M, 5BB, and Beta showed superior grafting performance, with scion germination and mating interface healing rates both reaching 90% or higher, and root callus formation exceeding 75% (Figure 1). High scion germination and graft union healing rates with 101-14M rootstock were also reported by Han et al. [29], consistent with our findings. Graft compatibility is closely linked to hormone signaling. Low auxin (IAA) concentrations may suppress abscisic acid (ABA) and anti-ethylene/antioxidant compound synthesis, thereby reducing graft incompatibility [30]. We therefore hypothesize that hormone levels—particularly IAA—in rootstock–scion combinations significantly influence grafting success, which requires further physiological experimentation. Additionally, the rootstock rooting rate was lower than other indicators, likely due to the ‘Fengguang’ scion’s genetics. In practice, growers typically compensate by applying rooting agents to improve root development and plant vigor. The larger scion TCSD induced by 5BB (Figure 2a,b) aligns with findings in ‘Petit Verdot’ [19], though SO4 showed greater vigor in ‘Summer Black’ [24], highlighting scion-specific responses.

The influence of rootstocks on yield varies by scion–rootstock combination. Reynolds and Wardle [31] found no significant yield differences between 5BB, SO4, 3309C, and 5C rootstocks and own-rooted vines across nine wine grape cultivars, whereas Jogaiah et al. [32] reported lower yields in ‘Thompson Seedless’ grafted on 1103P and Dogridge than own-rooted vines. In our study, SO4 produced the highest yield, while the lowest yield was produced by 101-14M, and both of them were significantly different from own-rooted vines (Table 1), consistent with findings in ‘Summer Black’ [24]. These apparent discrepancies might be primarily attributed to the distinct genetic background of the scion cultivars, which dictates their specific physiological responses to different rootstocks. Furthermore, the expression of rootstock effects is strongly modulated by local environmental conditions, particularly nutrient availability [33]. Therefore, the significant yield variation observed in our study underscores that rootstock performance is not absolute but is context-specific, shaped by the interplay of scion genotype and the growing environment.

The selection of an appropriate rootstock is critical for modulating berry quality traits. In the present study, SO4 rootstock yielded the highest cluster mass (Table 1), aligning with previous observations in ‘Summer Black’ grapes [23]. However, the influence of rootstock on berry mass was inconsistent across scion cultivars: while 101-14M significantly increased berry mass compared to own-rooted vines in this study, it led to a reduction in ‘Chunguang’ [34] and showed no significant effect in ‘Gold Finger’ [35]. Such genotype-specific responses support the concept that graft performance is governed by a complex scion–rootstock interaction rather than the rootstock’s intrinsic effect alone, as highlighted by Tandonnet et al. [36]. This interaction likely involves differential gene expression and hormonal signaling between rootstock and scion [30,37], ultimately shaping phenotypic outcomes in a cultivar-specific manner. In terms of postharvest potential, vines on SO4 exhibited significant reductions in pulling resistance, pressure resistance, and flesh firmness relative to own-rooted vines (Table 2), a trend also noted in SO4-grafted ‘Chunguang’ vines [34]. These results implied a consistent negative impact of SO4 on mechanical properties across cultivars. Moreover, a notable decrease in total soluble solids (TSS) was observed in vines grafted onto Beta, 5C, SO4, and 5BB (Table 2), consistent with earlier findings in ‘Summer Black’ [21]. This general decline in TSS may reflect a shift in carbon partitioning or water uptake dynamics induced by the rootstocks [38], requiring further physiological investigation.

**Figure 4:**
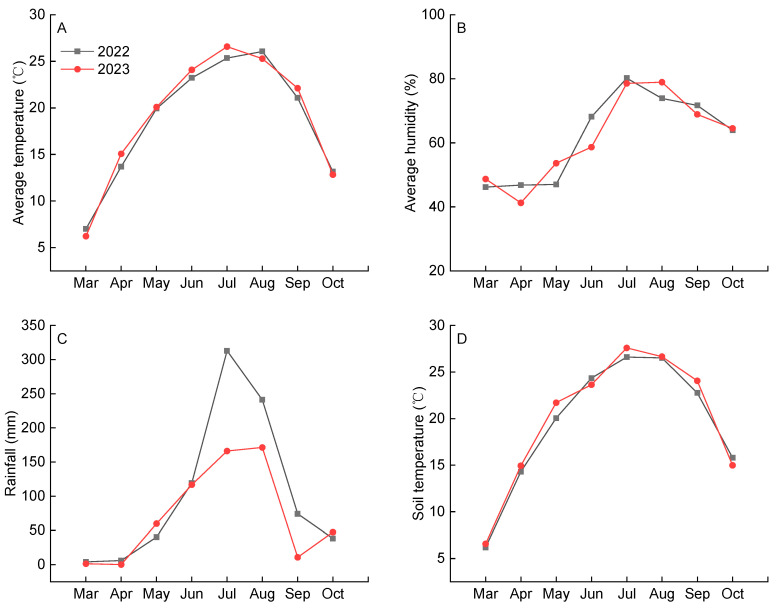
Monthly average temperature, humidity, rainfall, and soil temperature (at a depth of 20 cm) during the growing season in 2022 and 2023. (**A**) Average temperature; (**B**) Average humidity; (**C**) Rainfall; (**D**) Soil temperature.

An inverse relationship was observed between vegetative growth and yield across the two study years. The higher shoot growth in 2022, compared to 2023, contrasted with the lower yield that year (Figure 2, Table 1). This pattern may be attributed to greater rainfall during the key growth period (June–September) of 2022 (Figure 4C), which likely favored vegetative development over reproductive output. The PCA showed 101-14M had advantages in fruit quality but lower yield, while SO4 showed the opposite (Figure 3), demonstrating a balance between yield and fruit quality. The comprehensive ranking placed 101-14M first and SO4 last (Table 3), indicating that high yield alone was insufficient to compensate for poor fruit quality. This outcome diverges from the optimal rootstock (3309C) identified for ‘Petit Verdot’ [19], highlighting that optimal rootstock selection is highly scion-specific.

## 4. Materials and Methods

### 4.1. Experimental Site and Growth Conditions

The experiment was conducted in an experimental vineyard located in Shigezhuang, Chang li City, northeast of Hebei Province, China (39°45′12″ N, 119°12′23″ E, altitude 20 m above sea level), within two production cycles in 2022–2023. The religion is recognized as one of the main producing areas of table grapes in China, with a semi-humid continental climate. The meteorological data of the entire growing season during the experiment were presented in Figure 4. The soil is classified as sandy loam, and its detailed nutritional properties are provided in Table 4.

### 4.2. Plant Materials, Experimental Design and Vine Management

The table grape ‘Fengguang’ was selected as the scion cultivar, which originated from a hand-pollinated cross between ‘Kyoho’ and ‘Muscat Hamburg’ conducted in 2003 [13]. It is characterized by large berries, high sugar content, labor-saving management, and rich strawberry fragrance. The 100 mature scions of table grape ‘Fengguang’ were grafted on hardwood cuttings of eight resistant rootstocks (101-14M, 110R, 188-08, 3309C, 5BB, 5C, SO4 and Beta) and nongrafted vines, defined as own-rooted vines, were used as control. The resistance characteristics of 8 rootstocks, as summarized from current studies [39,40,41,42,43], are presented in Table 5. Mature scions were collected from healthy, one-year-old shoots of 4-year-old, self-rooted ‘Fengguang’ vines, and these grafted plants were then established at the Shigezhuang experimental station. The grafting experiment was conducted in April 2018. Both grafted and own-rooted plants were initially grown in a nursery for one month before being transplanted to the field. Vines were trained in a pergola system at a height of 1.8 m, with plant spacing of 0.8 m × 3.0 m. During the growing season, 8–10 fruit-bearing new shoots were pruned per vine and only one fruit cluster was retained on each shoot. A balanced fertilization protocol was applied throughout the growth cycle: 30,000 kg ha^−1^ organic fertilizer, 200 kg ha^−1^ urea, 150 kg ha^−1^ phosphorus pentoxide (P_2_O_5_) and 200 kg ha^−1^ potassium oxide (K_2_O). Additionally, a foliar spray of a 1% borax solution (150 kg ha^−1^) was applied 7–10 days before flowering to enhance reproductive development. Drip irrigation was applied according to the phenological period. During winter pruning, two-bud spurs were retained per cane. The experiment was arranged in a completely randomized block design with three replications. Each replication consists of six vines, with three guard vines positioned at the end of each block to minimize edge effects. Graft compatibility indices of scion–rootstock combinations were evaluated in 2018 (year of establishment). Growth, yield, and fruit quality were measured from 2022 to 2023.

### 4.3. Affinity Metrics for Grafting

From each rootstock–scion combination, 80 randomly selected grafted segments (post-rooting) were divided into four equal groups. The following parameters were then evaluated: the germination rate of scions, healing rate of the mating interface, generation rate of root callus and rooting rate of rootstocks. The calculation formulas for these parameters were standardized as follows:

$$\mathrm{The}\;\mathrm{germination}\;\mathrm{rate}\;\mathrm{of}\;\mathrm{scions}\;(\%)\;=\;\frac{Number\;of\;grafted\;scions}{Total\;number\;of\;investigations}\times 100\%$$

$$\mathrm{The}\;\mathrm{healing}\;\mathrm{rate}\;\mathrm{of}\;\mathrm{the}\;\mathrm{mating}\;\mathrm{interface}\;(\%)=\frac{Number\;of\;interface\;heals}{Total\;number\;of\;investigations}\times 100\%$$

$$\mathrm{The}\;\mathrm{generation}\;\mathrm{rate}\;\mathrm{of}\;\mathrm{root}\;\mathrm{callus}\;(\%)=\frac{Number\;of\;root\;calluses}{Total\;number\;of\;investigations}\times 100\%$$

$$\mathrm{The}\;\mathrm{rooting}\;\mathrm{rate}\;\mathrm{of}\;\mathrm{rootstocks}\;(\%)=\frac{Number\;of\;rooted\;rootstocks}{Total\;number\;of\;investigations}\times 100\%$$

### 4.4. Growth Parameters

Scion trunk cross-sectional diameter (the longest axial dimension) was measured at a standardized height of 20 cm above the graft union, and shoot basal diameter was recorded at the basal internode (the longest axial dimension) using digital vernier calipers.

### 4.5. Yield Calculation

The average cluster weight (g) and the numbers of fruit-bearing branches per vine were quantified during the harvest period. Vine yield (kg/vine) was calculated as the product of these two yield components.

### 4.6. Fruit Quality Parameters

The table cultivar ‘Fengguang’ was harvested manually at 102 days after anthesis (DAA), based on commercial maturity standards (seeds turned brown). Two clusters were randomly sampled from each vine. Fifteen berries were randomly picked from the shoulder, the middle part, and the tail of each cluster. These fresh berries were used to determine fruit quality indicators, with all measurements performed in three replicates. Cluster mass was measured with 5 clusters per replicate. Berry mass was determined by weighing 30 berries and dividing the total weight by 30 for each replicate. Cluster mass and berry mass were weighed with an electronic balance. Measurements of berry weight, vertical diameter (BV), horizontal diameter (BH), pulling resistance, pressure resistance, and flesh firmness were conducted using 10 berries per replicate. BV and BH were measured with a digital caliper and the berry shape index was calculated as BV/BH. Pulling resistance and pressure resistance were determined using a fruit pressure-tension tester (NK-30, Algol Instrument, Co., Ltd., Taiwan, China). Flesh firmness was measured with a fruit hardness tester (KM-1, Takemura Electric Works, Tokyo, Japan) equipped with a 5 mm-diameter cylinder tip. Thirty randomly selected berries per replicate were juiced using a fresh-squeeze juicer for the analysis of the total soluble solids (TSS) and titratable acid (TA). The TSS (%) and TA (%) were determined with a digital refractometer (PAL-1, Atago, Tokyo, Japan) and fruit acidity meter (GMK-835F, G-WON Hitech Co., Ltd., Seoul, Republic of Korea), respectively. TA was expressed as percent citric acid equivalent. The ratio of sugar to acid was expressed as TSS/TA.

### 4.7. Statistical Analysis

Data analysis was performed using SPSS 26.0 (IBM, Chicago, IL, USA). Mean values were compared by one-way analysis of variance (ANOVA), followed by Duncan’s test at *p* < 0.05. The means of all the tested yield and quality attributes were analyzed by the principal components analysis (PCA) using Origin 2022 (OriginLab Corporation, Northampton, MA, USA). Additionally, a comprehensive evaluation of yield and fruit quality across different rootstock–scion combinations were carried out using the subordinate function value method [44,45]. This method assesses each index for every treatment, followed by accumulating the individual index evaluations to derive an overall evaluation value.

The membership function value was calculated using Formula (1):

(1) $${X^{\prime }}_{ij}=\frac{X_{ij}-\min\left(X_{j}\right)}{\max\left(X_{j}\right)-\min\left(X_{j}\right)}\times \alpha +\left(1-\alpha \right)\;\;\;(i=1,2,\ldots ,m;j=1,2,\ldots ,n)\;$$

${X^{’}}_{ij}$ represented the membership function value. $X_{ij}$ was the raw data value of the *j*-th index from the *i*-th treatment. $\min\left(X_{j}\right)$ and $\max\left(X_{j}\right)$ were the minimum and maximum value of the *j*-th index; α was the efficacy coefficient (α= 0.6).

Weight value was calculated using Formulas (2)–(4):

(2) $$f_{ij}=\frac{{X^{\prime }}_{ij}}{\sum_{i=1}^{m}{X^{\prime }}_{ij}},\;i=1,2,\ldots ,m;j=1,2,\ldots ,n\;\;\;\;$$

(3) $$H_{j}=-\frac{1}{lnm}\sum_{i=1}^{m}f_{ij}lnf_{ij}\;\;\;\;$$

(4) $$W_{j}=\frac{1-H_{j}}{n-\sum_{j=1}^{n}H_{j}}\;\;\;\;\;\;$$

$H_{j}$ represented the entropy value of the *j*-th index. $W_{j}$ was the weight value of *j*-th index.

The comprehensive evaluation value was calculated using Formula (5):

(5) $$R_{i}=\sum_{j=1}^{n}{X\prime }_{ij}W_{j}$$

$R_{i}$ represented the comprehensive evaluation value. The comprehensive results were ordered based on the evaluation value.

## 5. Conclusions

Rootstocks significantly influenced graft compatibility, growth vigor, yield, and fruit quality in ‘Fengguang’ vines. Vines on 101-14M, 5BB, and Beta exhibited good performance in graft compatibility indicators (excluding the rooting rate of rootstocks). Vines on 5BB showed strong growth vigor, which was statistically equivalent to own-rooted vines. Compared to own-rooted vines, SO4 exhibited superior performance in yield and cluster mass and 101-14M demonstrated better traits related to fruit quality (including berry mass, berry vertical diameter, pressure resistance, flesh firmness). Based on a comprehensive evaluation of yield and fruit quality attributes, the rootstock–scion combinations were ranked as follows: 101-14M, own root, Beta, 110R, 5C, 5BB, 188-08, 3309C, SO4. 101-14M was the most effective in improving the fruit quality of ‘Fengguang’ vines and therefore highly recommended for commercial graft-cultivation of this cultivar.

The findings of this study, based on two consecutive growing seasons (2022–2023), offer valuable initial insights. Nevertheless, the long-term stability of yield and fruit quality across variable climatic conditions, along with the economic sustainability of these combinations, remains to be fully evaluated. Consequently, future work should prioritize multi-annual and multi-location trials and mechanistic investigations to validate and extend these results. Furthermore, these pronounced phenotypic differences provide a critical and reliable foundation for future research aimed at elucidating the underlying biochemical mechanisms, including the modulation of biologically active compounds by rootstock selection.

## Figures and Tables

**Table 1:** Impacts of different rootstocks on yield and appearance attributes of cluster and berry of ‘Fengguang’ grapes.

Year	Rootstock	Yield (kg/vine)	Cluster Mass (g)	Berry Mass (g)	BV ^1^ (mm)	BH ^2^ (mm)	BV/BH
2022	101-14M	4.31 ± 0.08 e ^3^	529.53 ± 10.32 c	12.32 ± 0.15 a	31.60 ± 1.86 a	27.00 ± 2.11 a	1.17 ± 0.05 abc
	110R	4.98 ± 0.19 b	547.27 ± 20.53 bc	11.48 ± 0.09 c	29.85 ± 2.21 bc	25.20 ± 1.46 abc	1.18 ± 0.06 ab
	188-08	4.71 ± 0.06 cd	540.53 ± 7.33 bc	11.02 ± 0.04 f	28.84 ± 1.85 bc	24.21 ± 1.54 c	1.19 ± 0.09 a
	3309C	4.81 ± 0.27 bcd	526.77 ± 29.10 c	10.73 ± 0.09 g	28.24 ± 1.58 c	25.00 ± 1.69 bc	1.13 ± 0.04 bc
	5BB	4.65 ± 0.13 d	559.27 ± 14.82 b	11.13 ± 0.05 ef	29.10 ± 1.99 bc	25.07 ± 1.74 bc	1.16 ± 0.06 abc
	5C	4.91 ± 0.05 bc	539.23 ± 5.59 bc	11.86 ± 0.07 b	30.22 ± 1.24 ab	26.61 ± 2.00 ab	1.14 ± 0.06 abc
	SO4	5.47 ± 0.02 a	585.67 ± 1.86 a	10.52 ± 0.12 h	28.09 ± 2.60 c	25.23 ± 2.43 abc	1.11 ± 0.06 c
	Beta	5.04 ± 0.06 b	561.93 ± 6.71 ab	11.20 ± 0.03 de	29.51 ± 1.62 bc	25.88 ± 1.32 abc	1.14 ± 0.05 abc
	Own root	4.81 ± 0.05 bcd	557.13 ± 4.92 b	11.32 ± 0.04 d	29.71 ± 1.26 bc	26.34 ± 1.52 ab	1.13 ± 0.05 bc
2023	101-14M	4.59 ± 0.23 d	551.30 ± 28.05 c	11.95 ± 0.10 a	29.94 ± 1.94 a	25.97 ± 1.79 a	1.16 ± 0.12 ab
	110R	5.04 ± 0.22 bc	569.87 ± 25.12 bc	11.35 ± 0.07 c	27.83 ± 1.85 cd	24.50 ± 1.48 b	1.14 ± 0.07 ab
	188-08	4.80 ± 0.10 cd	560.92 ± 11.37 bc	10.93 ± 0.10 e	29.29 ± 1.79 abc	24.45 ± 1.14 b	1.20 ± 0.04 a
	3309C	4.99 ± 0.15 bc	560.40 ± 16.65 bc	10.67 ± 0.09 f	29.60 ± 1.69 ab	25.53 ± 1.35 ab	1.16 ± 0.06 ab
	5BB	4.87 ± 0.08 bc	589.47 ± 9.78 ab	10.97 ± 0.07 e	28.22 ± 2.10 bcd	24.66 ± 1.20 b	1.15 ± 0.04 ab
	5C	5.15 ± 0.12 b	578.30 ± 13.00 abc	11.74 ± 0.08 b	28.23 ± 1.71 bcd	25.06 ± 1.19 ab	1.13 ± 0.04 b
	SO4	5.60 ± 0.16 a	607.90 ± 17.95 a	10.39 ± 0.11 g	27.28 ± 1.51 d	24.40 ± 1.66 b	1.12 ± 0.05 b
	Beta	5.11 ± 0.09 b	578.97 ± 9.52 abc	11.15 ± 0.06 d	29.11 ± 1.18 abc	25.53 ± 0.88 ab	1.14 ± 0.05 ab
	Own root	4.94 ± 0.14bc	576.70 ± 16.23 abc	11.22 ± 0.03 cd	29.34 ± 0.67 abc	25.45 ± 0.61 ab	1.15 ± 0.04 ab

^1^ BV, berry vertical diameter. ^2^ BH, berry horizontal diameter. ^3^ Values represent mean ± SE. Different lowercase letters within each column indicate significant differences at *p* < 0.05 by Duncan’s test.

**Table 2:** Impacts of different rootstocks on physical and chemical indicators of ‘Fengguang’ grapes.

Year	Rootstock	Pulling Resistance (N)	Pressure Resistance (N)	Flesh Firmness (kg/cm^2^)	TSS ^1^ (%)	TA ^2^ (%)	TSS/TA
2022	101-14M	4.84 ± 0.26 a ^3^	19.40 ± 0.61 a	0.70 ± 0.03 a	17.57 ± 0.35 a	0.72 ± 0.02 bc	24.51 ± 0.07 a
	110R	4.79 ± 0.14 ab	18.08 ± 0.36 cde	0.54 ± 0.02 e	17.13 ± 0.15 bc	0.73 ± 0.02 ab	23.37 ± 0.27 b
	188-08	4.62 ± 0.18 bc	17.86 ± 0.45 de	0.62 ± 0.02 b	17.03 ± 0.21 bc	0.73 ± 0.02 abc	23.44 ± 0.29 b
	3309C	4.83 ± 0.20 a	17.77 ± 0.29 e	0.62 ± 0.02 b	16.80 ± 0.30 cd	0.70 ± 0.02 c	23.89 ± 0.41 ab
	5BB	4.77 ± 0.14 abc	18.37 ± 0.27 c	0.61 ± 0.02 bc	16.40 ± 0.36 de	0.75 ± 0.02 a	21.97 ± 0.28 c
	5C	4.61 ± 0.10 c	19.19 ± 0.38 a	0.56 ± 0.01 d	16.73 ± 0.21 cd	0.70 ± 0.02 c	23.80 ± 0.41 ab
	SO4	4.30 ± 0.18 d	17.24 ± 0.30 f	0.50 ± 0.03 f	16.27 ± 0.21 e	0.74 ± 0.02 ab	22.10 ± 0.80 c
	Beta	4.64 ± 0.15 bc	18.16 ± 0.26 cd	0.62 ± 0.02 b	16.87 ± 0.12 c	0.75 ± 0.01 a	22.59 ± 0.33 c
	Own root	4.72 ± 0.15 abc	18.73 ± 0.42 b	0.59 ± 0.02 c	17.40 ± 0.10 ab	0.71 ± 0.01 bc	24.40 ± 0.10 a
2023	101-14M	4.82 ± 0.24 a	21.01 ± 1.14 a	0.79 ± 0.05 a	18.03 ± 0.12 a	0.71 ± 0.02 c	25.29 ± 0.69 a
	110R	4.78 ± 0.17 a	18.80 ± 0.79 b	0.56 ± 0.03 cd	17.20 ± 0.10 cde	0.70 ± 0.02 c	24.70 ± 0.46 ab
	188-08	4.38 ± 0.19 d	17.64 ± 0.32 d	0.61 ± 0.02 b	17.30 ± 0.17 cd	0.73 ± 0.02 abc	23.82 ± 0.45 bc
	3309C	4.77 ± 0.13 ab	17.32± 0.20 de	0.56 ± 0.03 cd	17.43 ± 0.21 bc	0.72 ± 0.03 bc	24.11 ± 0.68 b
	5BB	4.74 ± 0.15 ab	17.29 ± 0.23 de	0.58 ± 0.02 bc	16.90 ± 0.10 f	0.75 ± 0.02 ab	22.44 ± 0.38 d
	5C	4.54 ± 0.13 c	18.22 ± 0.30 c	0.54 ± 0.02 d	17.13 ± 0.06 def	0.71 ± 0.02 c	24.14 ± 0.76 b
	SO4	4.20 ± 0.16 e	17.06 ± 0.31 e	0.48 ± 0.04 e	16.93 ± 0.15 ef	0.76 ± 0.03 a	22.30 ± 0.60 d
	Beta	4.61 ± 0.15 bc	17.62 ± 0.24 d	0.58 ± 0.02 bc	17.20 ± 0.10 cde	0.75 ± 0.01 ab	22.94 ± 0.39 cd
	Own root	4.66 ± 0.13 abc	17.64 ± 0.28 d	0.56 ± 0.02 cd	17.67 ± 0.21 b	0.72 ± 0.02 bc	24.43 ± 0.23 ab

^1^ TSS, the total soluble solids. ^2^ TA, titratable acid. ^3^ Values represent mean ± SE. Different lowercase letters within each column indicate significant differences at *p* < 0.05 by Duncan’s test.

**Table 3:** Comprehensive evaluation of different rootstocks on yield and berry quality of ‘Fengguang’.

Rootstock	YL	CM	BM	BV	BH	BV/BH	PU	PR	FF	TSS	TA	TSS/TA	Comprehensive Evaluation Value	Rank
101-14M	0.03	0.03	0.08	0.08	0.08	0.06	0.08	0.07	0.07	0.07	0.04	0.07	0.76	1
110R	0.06	0.05	0.06	0.05	0.04	0.06	0.07	0.05	0.04	0.05	0.04	0.06	0.61	4
188-08	0.05	0.04	0.04	0.05	0.03	0.08	0.05	0.04	0.05	0.05	0.05	0.05	0.58	7
3309C	0.05	0.03	0.04	0.05	0.05	0.05	0.07	0.04	0.05	0.05	0.04	0.06	0.57	8
5BB	0.05	0.06	0.05	0.04	0.04	0.05	0.07	0.04	0.05	0.03	0.08	0.03	0.59	6
5C	0.06	0.05	0.07	0.05	0.06	0.04	0.06	0.05	0.04	0.04	0.03	0.06	0.60	5
SO4	0.08	0.08	0.03	0.03	0.04	0.03	0.03	0.03	0.03	0.03	0.07	0.03	0.51	9
Beta	0.06	0.06	0.05	0.05	0.06	0.04	0.06	0.04	0.05	0.05	0.07	0.04	0.63	3
Own root	0.05	0.05	0.05	0.06	0.06	0.05	0.07	0.05	0.04	0.06	0.04	0.06	0.65	2

Abbreviations: See Figure 3 for details.

**Table 4:** The nutrient status of the soil within the 0–60 cm depth profile.

Depth	Organic Matter (%)	Total N(%)	Available P (mg/kg)	Available K (mg/kg)	Available Ca (mg/kg)	Available Mg (mg/kg)
0–20 cm	3.08	0.19	1.84	2.13	3.28	3.62
20–40 cm	2.63	0.12	1.05	1.94	2.04	3.57
40–60 cm	1.66	0.13	0.93	1.85	2.51	3.52

**Table 5:** Resistance of rootstocks to biotic and abiotic stresses.

Rootstocks	Phylloxera	Salinity	Drought	Coldness
101-14M	HR	R	S	R
110R	HR	MR	HR	LR
188-08	**–**	LR	MR	**–**
3309C	HR	MR	**–**	MR
5BB	HR	MR	S	HR
5C	HR	MR	**–**	MR
SO4	HR	S	R	HR
Beta	S	MR	S	HR

Responses to stress were categorized into resistance (R), high resistance (HR), moderate resistance (MR), low resistance (LR), and sensitivity (S), as described in the referenced studies.

## Data Availability

The datasets generated during this study are fully available within the article.
